# Clinicopathological and prognostic value of epithelial cell adhesion molecule in solid tumours: a meta-analysis

**DOI:** 10.3389/fonc.2023.1242231

**Published:** 2023-08-16

**Authors:** Peiwen Ding, Panyu Chen, Jiqi Ouyang, Qiang Li, Shijie Li

**Affiliations:** ^1^ Department of Oncology, Hospital of Chengdu University of Traditional Chinese Medicine, Chengdu, China; ^2^ Clinical School, Chengdu University of Traditional Chinese Medicine, Chengdu, China; ^3^ Operating Room, Sichuan University West China Hospital School of Nursing, Chengdu, China; ^4^ Department of Gastroenterology, China Academy of Chinese Medical Sciences Guang’anmen Hospital, Beijing, China

**Keywords:** EpCAM, solid tumors, prognosis, cancer stem cells, biomarkers, meta-analysis

## Abstract

**Background:**

Malignant tumors, mainly solid tumors, are a significant obstacle to the improvement of life expectancy at present. Epithelial cell adhesion molecule (EpCAM), a cancer stem cell biomarker, showed widespread expression in most normal epithelial cells and most cancers. Although the clinical significance of EpCAM in various malignant solid tumors has been studied extensively, the latent relationships between EpCAM and pathological and clinical characteristics in solid tumors and differences in the roles of EpCAM among tumors have not been clearly determined. The destination point of this study was to analyze the value of EpCAM in solid tumors in clinicopathological and prognostic dimension using a meta-analysis approach.

**Method and materials:**

A comprehensive and systematic search of the researches published up to March 7th, 2022, in PubMed, EMBASE, Web of Science, Cochrane library and PMC databases was performed. The relationships between EpCAM overexpression, clinicopathological characteristics, and survival outcomes were analyzed. Pooled hazard ratios (HRs) with 95% confidence intervals (CIs) and odds ratios (ORs) were estimated as indicators of the degree of correlation. This research was registered on PROSPERO (International prospective register of systematic reviews), ID: CRD42022315070.

**Results:**

In total, 57 articles and 14184 cases were included in this study. High EpCAM expression had a significant coherence with a poorer overall survival (OS) (HR: 1.30, 95% CI: 1.08–1.58, P < 0.01) and a worse disease-free survival (DFS) (HR: 1.58, 95% CI: 1.28–1.95, P < 0.01), especially of gastrointestinal tumors’ OS (HR: 1.50, 95% CI: 1.15–1.95, P < 0.01), and DFS (HR: 1.84, 95% CI: 1.52–2.33, P < 0.01). The DFS of head and neck tumors (HR: 2.33, 95% CI: 1.51–3.61, P < 0.01) was also associated with the overexpression of EpCAM. There were no positive relationships between the overexpression of EpCAM and sex (RR: 1.03, 95% CI: 0.99–1.07, P = 0.141), T classification (RR: 0.93, 95% CI: 0.82–1.06, P = 0.293), lymph node metastasis (RR: 0.85, 95% CI: 0.54–1.32, P = 0.461), distant metastasis (RR: 0.97, 95% CI: 0.84–1.10, P = 0.606), vascular infiltration (RR: 1.05, 95% CI: 0.85–1.29, P = 0.611), and TNM stage (RR: 0.93, 95% CI: 0.83–1.04, P = 0.187). However, the overexpression of EpCAM exhibited a significant association with the histological grades (RR: 0.88, 95% CI: 0.80–0.97, P < 0.01).

**Conclusion:**

Based on pooled HRs, the positive expression of EpCAM was totally correlated to a worse OS and DFS in solid tumors. The expression of EpCAM was related to a worse OS in gastrointestinal tumors and a worse DFS in gastrointestinal tumors and head and neck tumors. Moreover, EpCAM expression was correlated with the histological grade. The results presented pointed out that EpCAM could serve as a prognostic biomarker for gastrointestinal and head and neck tumors.

**Systematic review registration:**

https://www.crd.york.ac.uk/prospero, identifier CRD42022315070.

## 1 Introduction

Cancer, the first or second principal cause of death in most countries (1), is a significant obstacle to the improvement of life expectancy at present (2). Epithelial cell adhesion molecule (EpCAM), which showed widespread expression in most normal epithelial cells and most cancers, is an epithelial glycoprotein encoded by GA-733-2 (3). The molecule is participated in various physiological processes, such as cell adhesion, proliferation, migration, and mitotic signal transduction (4). EpCAM has been identified as a cancer stem cell (CSC) marker. CSCs have strong self-renewal ability and are directly related to tumor formation (5), accounting for 0.05–3% of the total number of tumor cells (6). CSCs have been a focus of research in recent decades and have been implicated in tumor generation, metastasis, recurrence, heterogeneity, resistance to chemotherapy and radiotherapy, and avoidance of immune surveillance (7, 8). A number of recent meta-analyses have shown that EpCAM expression levels are competent to serve as a significant prognostic marker in stomach (9), hepatic (10), prostate (11), and colorectal malignant tumors (12). Although the clinical significance of EpCAM in various cancers has been widely studied, differences of EpCAM expression in heterogeneous cancers and the relationships between EpCAM and pathological and clinical characteristics in solid tumors have not been determined. We employed a meta-analysis method for this investigation to comprehensively and systematically analyze EpCAM expression in different cancers and its relationship with survival outcomes and clinical characteristics. Furthermore, we conducted subgroup analyses to establish the prognostic and clinical validity of EpCAM in different cancers. The results provide basis for further studies of the applications of EpCAM.

## 2 Methods and materials

### 2.1 Search strategy and inclusion criteria

A comprehensive and systematic search of studies published up to March 7, 2022, in PubMed, EMBASE, Web of Science, Cochrane library and PMC databases was conducted. The search terms were as follows: (‘EpCAM’ OR ‘Epithelial Cell Adhesion Molecule’) AND (Tumor OR Neoplasm OR Neoplasia OR Cancer OR Carcinoma OR Malignancy) AND (Prognosis OR outcome OR survival).

Published articles that were in full compliance with following inclusion criteria were considered eligible: (1) published in English; (2) studies with pathologically accurate solid tumor diagnoses, including lung, breast, ovarian, gastric, hepatic, colorectal and pancreatic cancer and etc.; (3) EpCAM levels were detected by immunohistochemistry (IHC), quantitative real time-polymerase chain reaction (qRT-PCR), or enzyme-linked immunosorbent assay (ELISA); (4) studies evaluating the correlation between EpCAM overexpression and overall survival (OS), disease-free survival (DFS), and/or clinicopathological features of solid tumors; (5) hazard ratios (HRs) with 95% confidence intervals (CIs) are reported or data are available to calculate HRs and 95% CIs. Studies on the basis of the following criteria were excluded: (1) articles with overlapping or duplicate results, a lack of information, reviews, animal reports, conference abstracts, expert opinions, case reports, and letters; (2) studies irrelevant to the subjects of interest; (3) studies in which participators were in administration of any kind of anti-cancer treatment, for instance chemotherapy and radiotherapy, prior to surgical pathology or biopsy; (4) studies with a sample size of less than 40 patients.

### 2.2 Data extraction and quality assessment

Assessments of the abstract and the whole text, data retrieval, and data quality assessment were conducted by two researchers (PW Ding and PY Chen) independently. Key information was extracted into the baseline table. Differences that arose during the retrieval process were unraveled in reference with a third researcher. Extracted basic information contains: first author, year of publication, publication country, sample size, histological type, sampling method, EpCAM detection assay, cut-off value, follow-up time, HR estimation method, and HRs and 95% CIs. Patient characteristics included age, gender, histological grade, TNM stage, the size of tumor, T classification, lymphatic nodes metastasis, vascular invasion, and distant metastasis.

The HRs and 95% CIs of OS and DFS were obtained directly from the articles, when available. For studies that did not present HRs and 95% CIs, Kaplan-Meier survival curves (K-M curves) were used to estimate the results. The Newcastle-Ottawa Scale (NOS) was aimed to assess the qualities of included studies (13). An NOS score of 5 or higher indicated a high quality. Otherwise, the study was defined as low-quality. All processes were in observance of the PRISMA (Preferred Reporting Item for Systems Evaluation and Meta-Analysis) guidelines (14).

### 2.3 Statistical methods

Predictive capability of EpCAM overexpression for the prognosis of patients with solid tumors were appraised by the HRs and 95% CIs. Engauge Digitizer version 11.1 was applied to extract survival data from K-M curves, and STATA version 12.1 (STATA Corporation, College Station, TX, USA) was used for data processing of the meta-analysis.

When the heterogeneity in the combined studies was significant (I^2^> 60%), a random effects model was selected; otherwise, a fixed effect model was used. Furthermore,the sources of heterogeneity were determined by subgroup analyses. When estimating HR values, multivariate analyses adjusting for other prognostic factors were prioritized; otherwise, data from univariate analyses were used. K-M curves were used for the calculation of HRs (15, 16). Multivariate HR could better demonstrate the independent effect of high EpCAM expression in predicting the prognosis of patients with solid tumors. If the prognosis of patients with solid tumors with EpCAM over expression is poor, the combined HR should be more than 1.0, and its 95% CI should not overlap 1.0. Pooled odds ratios (ORs) were used to evaluate the relationship between clinicopathological characteristics and EpCAM positive expression. The continual deletion of individual studies was used to conduct a sensitivity analysis (17). To evaluate publication bias, a funnel plot, a Begg’s funnel plot, and the Egger test were utilized.

## 3 Results

### 3.1 Study inclusion and characteristics

**Figure 1:**
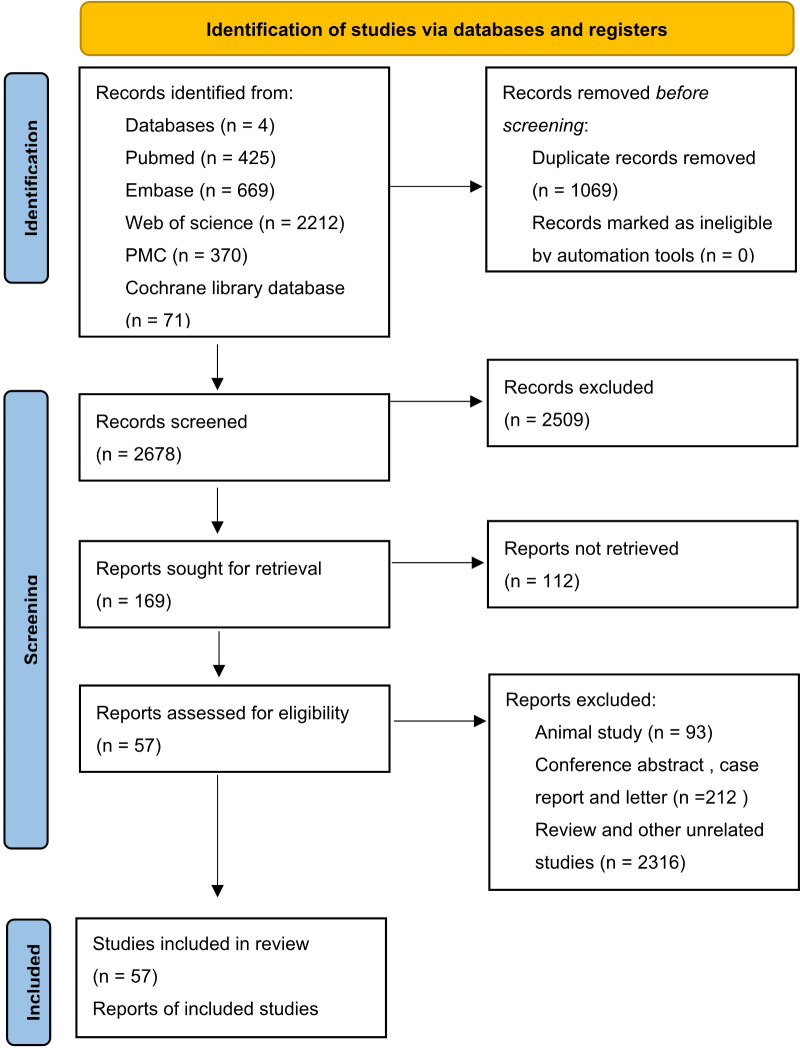
Flow chart of study identification process (14).

In total, 3747 studies in PubMed, EMBASE, Web of Science, Cochrane library and PMC databases were initially retrieved. After excluding 1069 duplications, 2678 studies were retained. Then, by sifting the titles and abstracts, 2509 studies were excluded. Of 169 full-text records evaluated in detail, 57 were in accordance with the exclusion and inclusion criteria and were selected for the final review (18–75). A flow diagram of researches selection is illustrated in **Figure 1**. The publication years ranged from 2000–2022. A total of 14184 cases were recruited in the selected studies. In detail, 12 out of 57 studies were in China, nine were in Germany, six each were in South Korea and Australia, five were in both Japan and the USA, and the others were in France, India, Iran, Netherlands, Romania, Sweden, Switzerland, Thailand, Turkey, the UK, and other countries. Additionally, 10 of 57 reports were focused on hepatocellular cancer, nine on breast cancer, four on head and neck squamous cell cancer, four on renal cell cancer, and the rest on lung, ampullary, colorectal, thyroid, cervical, pancreatic, and ovarian cancers (**Table 1**). There were 13 articles reporting only pathological features and 44 articles reporting both pathological features and prognostic results, including 36 articles targeting OS and 21 articles targeting DFS, RFS, or progression-free survival. Immunohistochemistry was a principal approach for detecting EpCAM, except for three articles using microarray, qRT-PCR, and ELISA as detection methods. Of note, in most articles, the total immunostaining score (TIS), which combines the proportion of staining with staining intensity, was used to divide the expression of EpCAM into high or low levels. Other articles used the positive percentage (pp), median expression, or a single factor, such as the intensity or the staining score as the cut-off value. All included articles were of high quality, with NOS scores of ranging five to eight.

**Table 1:** Main characteristics of 57 studies in the meta-analysis.

Author	Year	Country	Histological type	No	Sampling method	Method	Cut-off value	Outcome measures	NOS
Lin, Zhenzhen	2015	China	PTC	167	NA	IHC	NA	NA	6
Su, R.	2016	China	HCC	110	Surgery	IHC	TIS score>4	OS & RFS	8
Guo, Z.	2014	China	HCC	50	Surgery	IHC	PP>=5%	OS	7
Seino, S.	2018	Japan	HCC	251	surgery	IHC	PP>=5%	OS & RFS	7
Goossens-Beumer	2014	Netherland	CRC	309	surgery	IHC	expr>=88.5%	OS & dRFS	8
Chen, Xiao-Long	2016	China	GC	377	NA	IHC	NA	OS	7
AbdelMageed, Manar	2022	Sweden	CC	121	surgery	qRT-PCR	NA	DFS	7
Sundaram, S.	2020	India	BC	200	surgery	IHC	TIS score>5	NA	6
Kim, Y.	2009	Korea	NSCLC	234	surgery	IHC	TIS score>4	OS	6
Spizzo, G.	2003	Austria	BC	212	surgery	IHC	TIS score>4	OS & DFS	7
Pop, Miana Gabriela	2019	Romania	CC	80	surgery	IHC	TIS score>4	NA	8
Schinke, Henrik	2021	Germany	HNSCC	102	surgery	IHC	NA	OS	7
Piscuoglio, Salvatore	2012	Switzerland	AC	125	NA	IHC	PP>=5%	OS	7
Fong, D.	2008	Austria	PCA	153	NA	IHC	TIS score>4	OS	7
			AC	34	NA	IHC	TIS score>4	OS	
Stoecklein, N. H.	2006	Germany	ESCC	70	NA	IHC	Dako Score>=2	OS & RFS	7
Akita, H.	2011	Japan	PCA	95	surgery	IHC	TIS score>4	OS & DFS	7
Schmidt, M.	2011	Germany	BC	194	surgery	microarray	RNA median expression	DFS & MFS & DSS	8
Andriescu, Elena Corina	2019	Romania	PTC	70	surgery	IHC	TIS score>4	NA	8
Gao, Shuhang	2017	China	BC	134	NA	IHC	NA	NA	5
Noh, C. K.	2018	Korea	HCC	262	surgery	IHC	PP>=10%	OS & RFS	7
Agboola, A. J.	2012	UK	BC	726	NA	IHC	Histo-score	DFS	7
Soysal, S. D.	2013	USA	BC	1365	NA	IHC	Histo-score>99	OS	7
Yonaiyama, Shinnosuke	2013	Japan	IPMN	51	surgery	IHC	TIS score>4	OS	7
Seligson, D. B.	2004	USA	RCC	318	NA	IHC	PP>=5%	DSS	7
Sulpice, L.	2014	France	CCA	40	surgery	IHC	Intensity score>3	OS & DFS	8
Eichelberg, C.	2013	Germany	RCC	767	surgery	IHC	Staining score>=1	OS	7
Bae, J. S.	2012	Korea	HCC	175	NA	IHC	TIS score>3	OS	7
Piyathilake, C. J.	2000	USA	SCC	60	surgery	IHC	expression>=median	OS	7
Sen, S.	2016	India	OSCC	60	surgery	IHC	TIS score>4	NA	6
Went, P.	2005	Switzerland	RCC	182	NA	IHC	Staining score>=1	NA	7
Xu, M.	2014	China	HCC	106	surgery	IHC	MIOD>Median	OS & RFS	7
Chan, Anthony W. H.	2014	China	HCC	282	surgery	IHC	TIS score>4	OS & DFS	6
Went, P.	2006	Switzerland	CC/GC/PC/LC	1407	surgery	IHC	expr>70%	NA	6
Spizzo, G.	2004	Austria	BC	1715	surgery	IHC	TIS score>4	NA	6
Baumeister, Philipp	2018	Germany	HNSCC	94	NA	IHC	IHC score>median	OS	6
Sung, Jong Jin	2016	Korea	HCC	91	surgery	IHC	TIS score>4	NA	7
Zhou, N.	2015	China	LC	130	surgery	IHC	IRS>4	OS	7
Padthaisong, S.	2020	Thailand	CCA	178	surgery	IHC	TIS score>median	OS & RFS	7
Chen, Xin	2014	China	PA	74	surgery	IHC	TIS score>4	RFS	7
Spizzo, G.	2006	Austria	EOC	199	surgery	IHC	TIS score>4	OS	7
Chen, Xin	2014	China	Glioma	98	surgery	IHC	TIS score>4	OS	7
Varga, M.	2004	Austria	GBC	99	surgery	IHC	TIS score>4	OS	7
Ko, C. J.	2018	China	HCC	185	surgery	IHC	NA	OS	7
Battista, M. J.	2014	Germany	OC	117	surgery	IHC	NA	DSS & PFS	7
Woopen, H.	2014	Germany	EOC	74	surgery	IHC	expression>75%	OS	8
Dai, Xiao-Meng	2017	China	HCC	106	surgery	IHC	NA	OS & RFS	8
Gold, Kathryn A.	2014	USA	NSCLC	370	surgery	IHC	NA	OS & RFS	7
Bayram, Ali	2015	Turkey	HNSCC	60	surgery	IHC	staining>25%	NA	7
Schmidt, M.	2009	Germany	BC	402	surgery	IHC	TIS score>4	DFS	7
Spizzo, G.	2002	Austria	BC	205	surgery	IHC	TIS score>4	NA	7
Shim, H. S.	2009	Korea	OC	72	surgery	IHC	TIS score>4	OS	7
Murakami, N.	2021	Japan	Glottic Cancer	88	surgery	IHC	NA	OS & PFS	7
Murakami, N.	2019	Japan	HNSCC	100	Biopsy	IHC	NA	OS & PFS	7
Gebauer, Florian	2014	Germany	PDAC	66	Serum	ELISA	0.442 ng/ml	OS	8
Pak, M. G.	2012	Korea	NSCLC	164	surgery	IHC	2+ > 70%/3+ ≥30%	NA	7
Kalantari, Elham	2022	Iran	CRC	458	surgery	IHC	H-score>196	NA	8
Kim, H. L.	2005	USA	RCC	150	surgery	IHC	NA	DSS	7

PTC, Papillary thyroid carcinoma; HCC, Hepatocellular carcinoma; CRC, Colorectal cancer; GC, Gastric cancer; CC, Colon cancer; BC, Breast carcinoma; NSCLC, Non-small cell lung cancer; HNSCC, Head and neck squamous cell carcinoma; AC, Tumors of the ampulla of vater; PCA, Pancreatic and ampullary carcinomas; ESCC, Esophageal squamous cell carcinoma; IPMN, Intraductal papillary mucinous neoplasms; RCC, Renal cell carcinoma; CCA, Intrahepatic cholangiocarcinoma; SCC, Squamous cell cancer; OSCC, Oral squamous cell carcinoma; PC, Prostate cancers; LC, lung cancer; PA, Pituitary adenomas; EOC, Epithelial ovarian cancer; GBC, Gallbladder carcinoma; OC, Ovarian cancer; PDAC, Pancreatic adenocarcinoma; PP, percentage of positive cells ;TIS, the immunostaining score ( sum of staining intensity scores and percentage of positive cells scores ); exp, expression; MIOD, the mean integrated optical density; OS, overall survival; DFS, disease-free survival; RFS, relapse-free survival; DSS, disease-specific survival; PFS, progression-free survival; NA, not available.

### 3.2 Association between EpCAM and clinicopathological features

#### 3.2.1 EpCAM overexpression and OS

**Figure 2:**
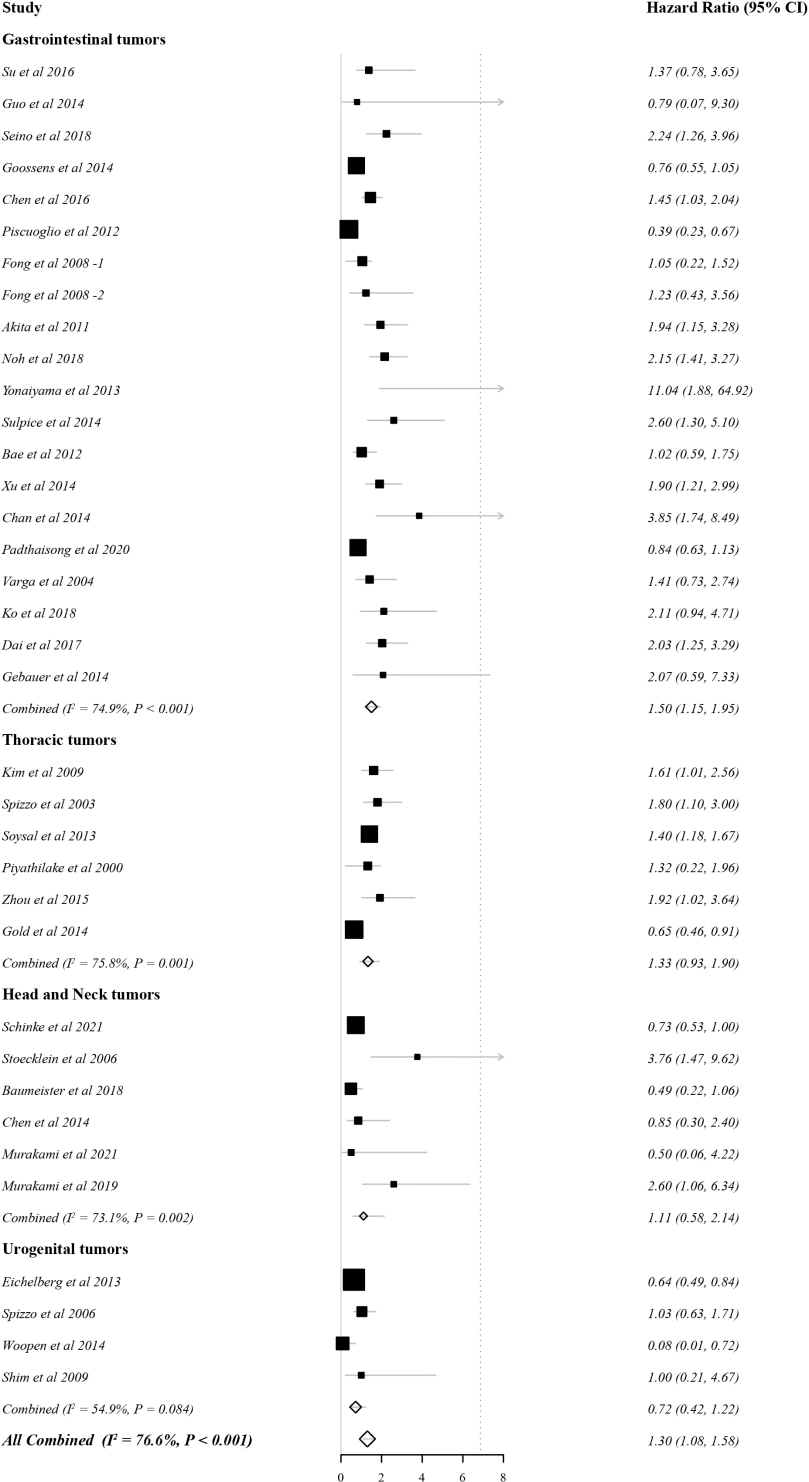
Forest plot of the effect of EpCAM status on overall survival.

Since there was obvious heterogeneity (76.6%; P < 0.01), the HRs and 95% CIs were evaluated by a random effects model. As shown in **Figure 2**, high EpCAM expression was significantly associated with a worse OS (HR: 1.30, 95% CI: 1.08–1.58, P < 0.01). In gastrointestinal tumors (HR: 1.50, 95% CI: 1.15–1.95, P < 0.01), the overexpression of EpCAM was significantly related to a worse OS. In thoracic tumors (HR 1.33, 95% CI: 0.93–1.90, P = 0.16) and head and neck tumors (HR: 1.11, 95% CI: 0.58–2.14, P = 0.752), the relationship was not significant. However, in urogenital tumors (HR = 0.70, 95% CI: 0.55–0.89, P = 0.22), the opposite relationship was detected.

#### 3.2.2 EpCAM overexpression and DFS

**Figure 3:**
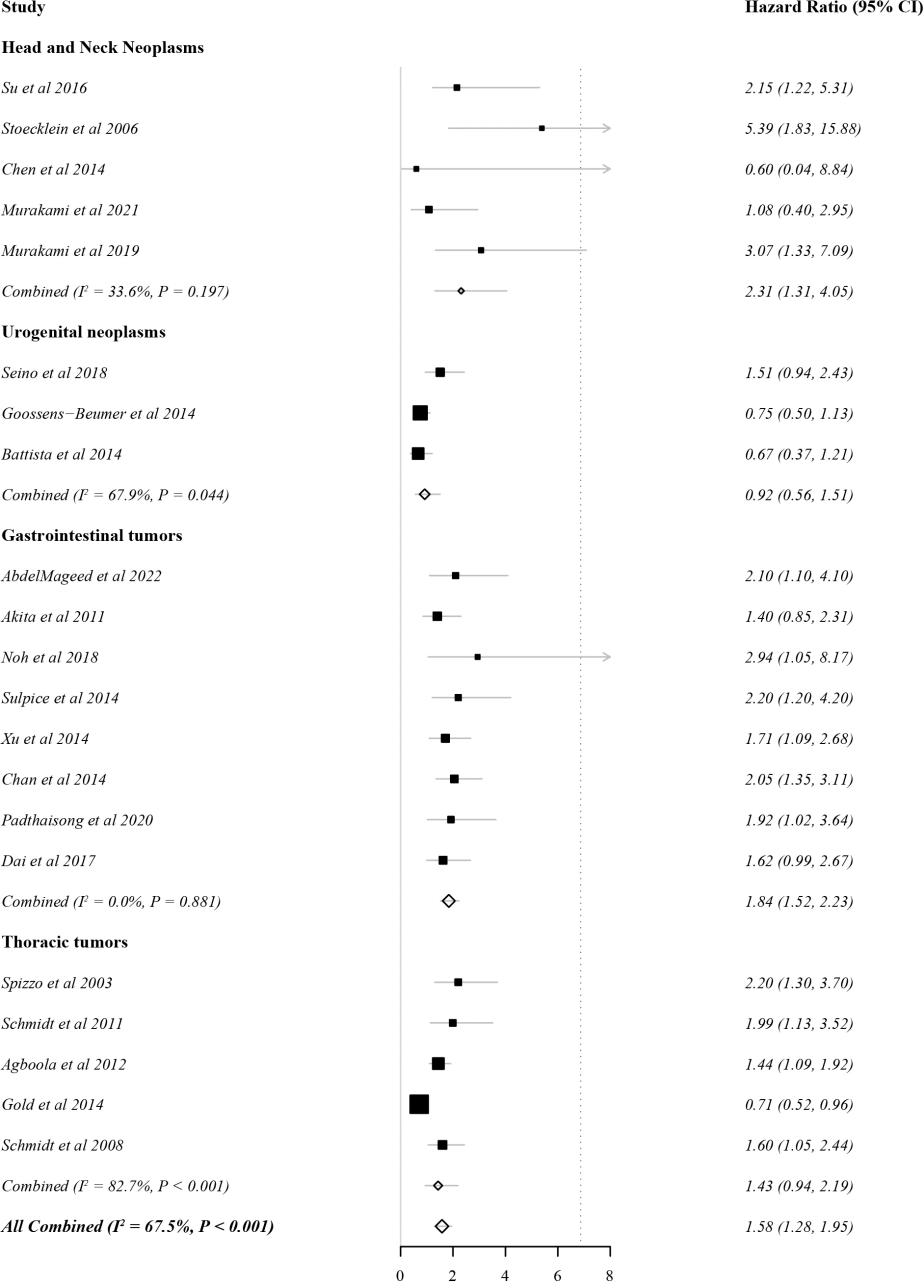
Forest plot of the effect of EpCAM status on disease-free survival.

In total, 21 studies evaluated DFS as the outcome indicator. Because heterogeneity was 67.5% (>60%), the HR and 95% CI were analysed by a random effects model. There was a significant correlation between EpCAM expression and a worse DFS (**Figure 3**, HR: 1.58, 95% CI: 1.28–1.95, P < 0.01). In gastrointestinal tumors (HR: 1.84, 95% CI: 1.52–2.33, P < 0.01) and head and neck tumors (HR: 2.33, 95% CI: 1.51–3.61, P < 0.01), the overexpression of EpCAM was significantly associated with a worse DFS. However, this relationship was unclear in thoracic tumors (HR 1.43, 95% CI: 0.94–2.19, P = 0.10) and urogenital tumors (HR 0.92, 95% CI: 0.56–1.51, P = 0.74).

#### 3.3 EpCAM overexpression: Pathological and clinical characteristics

EpCAM overexpression was not significantly related to sex (RR: 1.03, CI: 0.99–1.07, P = 0.141), T classification (RR: 0.93, CI: 0.82–1.06, P = 0.293), lymph node metastasis (RR: 0.85, CI: 0.54–1.32, P = 0.461), distant metastasis (RR: 0.97, CI: 0.84–1.10, P = 0.606), vascular infiltration (RR: 1.05, CI: 0.85–1.29, P = 0.611), and TNM stage (RR: 0.93, CI: 0.83–1.04, P = 0.187). However, it was significantly correlated with the histological grade (RR: 0.88, CI: 0.80–0.97, P < 0.01). The detailed information is listed in **Table 2**. Owing to a lack of data, the relationships between the overexpression of EpCAM and other pathological features were not explored.

**Table 2:** Results of the associations of high EpCAM expression with multiple clinicopathological parameters.

Categories	Studies (N)	OR (95% CI)	*P*-value	Heterogeneity		
				I^2^(%)	*P*-value	Model
Sex (male *vs.* female)	29	1.03 (0.99, 1.07)	0.141	44.9	0.005	Fixed
Histologic grade (well/moderately *vs.* poor)	35	0.88 (0.80, 0.97)	0.009	84.9	<0.001	Random
T classification (T1-2 *vs.* T3-4)	25	0.93 (0.82, 1.06)	0.293	60.2	0.004	Random
Lymph node metastasis (yes *vs.* no)	22	0.85 (0.54, 1.32)	0.461	96.7	<0.001	Random
Distant metastasis (yes *vs.* no)	13	0.97 (0.84, 1.10)	0.606	12.1	0.326	Fixed
TNM stage(I/II *vs.* III/IV)	19	0.93 (0.83, 1.04)	0.187	71.3	<0.001	Random
Vascular infiltration (yes *vs.* no)	12	1.05 (0.86, 1.29)	0.611	72.1	<0.001	Random

### 3.4 Sensitivity analysis and publication bias

**Figure 4:**
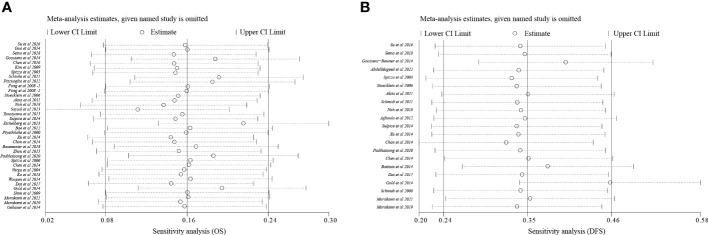
Sensitivity analysis. **(A)** Sensitivity analysis of OS; **(B)** Sensitivity analysis of DFS.

To analyze sensitivity, each study was successively deleted, revealing that the statistical result of relationships between EpCAM overexpression and survival periods were affected by no individual study. These outcomes attested to the validity of the meta-analysis (**Figure 4**).

**Figure 5:**
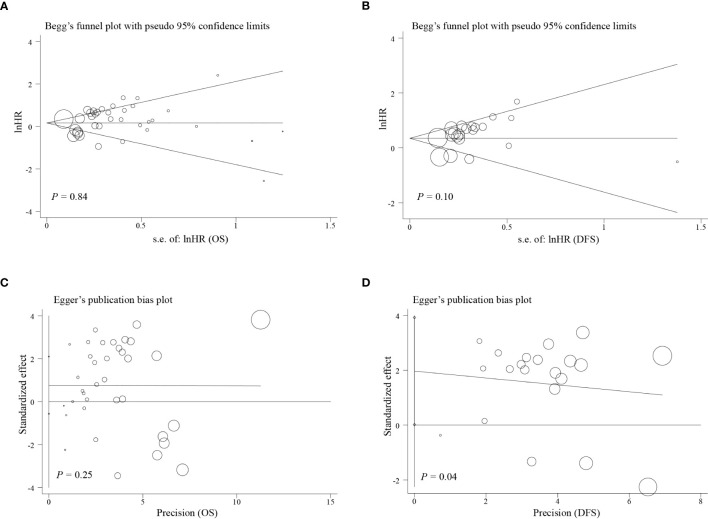
Funnel plot of the analysis on survival. **(A)** Begg’s funnel plot of the outcome of OS; **(B)** Begg’s funnel plot of the outcome of DFS; **(C)** Egger’s publication bias plot of outcome of OS; **(D)** Egger’s publication bias plot of the outcome of DFS.

Egger’s test, Begg’s test, and funnel plots were performed to make evaluation of publication bias. As illustrated in **Figures 4**, **5**, significant publication bias was not detected for OS (P = 0.84) and DFS (P = 0.10) by Begg’s tests. By Egger’s test, no obvious publication bias for OS (P = 0.25) was found; however, significant publication bias was detected for DFS (P = 0.04). This publication bias might be due to the smaller sample size for DFS than for OS and unpublished research that has not been included also was contributing.

## 4 Discussion

EpCAM, one of the first CSC biomarkers, was first discovered in 1979 as a colorectal cancer secretory antigen recognized by humoral immunity (5). Based on its prominent role in adhesion structure and polarity, EpCAM was considered a cell adhesion molecule initially (76). However, the complexities of EpCAM functions have recently been determined. It is now recognized that EpCAM does not play a significant role in cell adhesion and migration (77) and affects downstream pathways by inhibiting nPKCs (78, 79).

Hematological neoplasms were not included in this study, given the differences in growth and metastasis and the lack of studies focused on hematological neoplasms with EpCAM as a prognostic marker. This study indicated that the overexpression of EpCAM in solid tumors suggests a worse OS and DFS. In a subgroup analysis, high EpCAM expression in gastrointestinal tumors was related to a worse OS, while the opposite relationship was obtained for urogenital tumors. High EpCAM expression levels in gastrointestinal tumors and head and neck tumors suggest a worse DFS. In digestive system tumors, EpCAM was osculate related to a poor prognosis. Elevated EpCAM expression in solid tumors suggests a worse degree of cancer cell differentiation. Previously, it has reported that EpCAM is a predictor of tumor metastasis (80); however, this was not supported by the discoveries of current studies. In the following discussion, we discuss a few key issues relate to our results.

EpCAM, as a humoral immune antigen found in carcinoma of colon cells, is of significantly close relationship to congenital tufting enteropathy, inflammatory bowel disease, and cholestatic liver injury, in addition to cancer (81–84). It has also been reported that EpCAM plays a certain role in the differentiation and regeneration of hepatobiliary cells (85). EpCAM regulates intestinal epithelial homeostasis via various signaling pathways, including ROCK and nPKCs (86, 87). Although there is no laboratory evidence to prove that EpCAM is involved in gastrointestinal cancer, there is some evidence supporting this relationship. EpCAM and claudin-7’s interaction may be answerable to the growth of tumors in colorectal cancer. (88, 89).

It has been claimed that the overexpression of EpCAM can inhibit the migration of ovarian cancer cells stimulated by EGF (90). Direct evidence for the relationship between EpCAM expression and urological tumors, such as renal cell carcinoma, has not been reported. The clinical significance of the overexpression of EpCAM may differ between urogenital tumors and gastrointestinal tumors. Based on the function of EpCAM in separating the mesoderm and endoderm and guiding endoderm differentiation (91), the digestive system generally originates from the endoderm and the genitourinary system primarily originates from the mesoderm; cells of the two embryonic layers have various differences, and these differences may explain why EpCAM has opposite effects in the two distinct cancers. However, comparative analyses of the effects of EpCAM in different embryonic cells are lacking; accordingly, further laboratory research is needed to resolve this issue.

Second, EpCAM was upregulated in undifferentiated P19 cells of mouse embryonic cancer (92), but downregulated in differentiated ones. EpCAM is crucial for preserving the pluripotency of embryonic stem cells. EpICD of EpCAM supports pluripotency by activating the transcription of reprogramming factors (93). However, its mechanism of action in somatic stem cells is unclear (94). EpCAM affected somatic reprogramming by related pathways or by forming complexes with other molecules. For example, EpCAM as well as claudin-7 complexes are essential for somatic reprogramming in both mice and humans. To improve pluripotent reprogramming, EpCAM complexes may promote Oct4 transcription while blocking the p53 and p21 pathways (95). EpEX/EpCAM may also lead to the nuclear translocation of hypoxia inducible factor 1a, via stimulating signal transducer and activator of transcription 3 (STAT3), thus enabling somatic reprogramming. To synthesize human induced pluripotent stem cells, EpEX/EpCAM when combined with Oct3/4 or Kruppel-like factor 4 is adequate (96). Since EpCAM serves as crucial for the maintenance of cell pluripotency, it may be overexpressed in cancer cells with low differentiation.

Finally, as EpCAM was initially recognized as a cell adhesion molecule, the molecule was expected to influence cell adhesion, migration, and other functions (86). However, it showed no significant effects on lymph node and distal metastasis in this study,. Early studies of EpCAM suggested that it could be considered a homophilic cell adhesion molecule because its ectopic expression in mouse fibroblasts and mouse breast cancer cells induced cell aggregation and separation and reduced invasive growth (97). Subsequent studies found that EpCAM disrupted the combination of E-cadherin and cytoskeleton and inhibited E-cadherin-mediated cell aggregation (98, 99). It is also possible that EpCAM is an antagonist of E-cadherin. These contradictory opposite make the functions of EpCAM in terms of adhesion unclear. It had also been pointed out that the adhesion and migration functions of EpCAM are not related to its adhesion functions. Instead, it can inhibit myosin activity by regulating nonclassical nPKCs to produce downstream cascade reactions, thereby avoiding the excessive activation of myosin, leading to unstable adhesion contact and a loss of calcium mucoprotein (86). In terms of migration, there are conflicting results for EpCAM and EMT (90, 91). The view that EMT is critical for cell migration and invasion has been repeatedly challenged in recent years. Current research suggests a dynamic collective invasion mode (100, 101), in which EpCAM plays a complex role. As a single index, EpCAM expression is not a powerful tool to predict tumor progression, recurrence, and metastasis. More research is needed to characterize the multifaceted roles of EpCAM.

This study had the following limitations. First, the object of the study was pan-solid tumors, and there was substantial heterogeneity. Second, the impact of radiotherapy and chemotherapy after surgery or biopsy on survival was not considered, and this is another source of heterogeneity. Third, in addition to differences in detection methods, the cut-off values varied. Most studies adopted a TIS score of >4 as the standard, while others adopted PP ≥ 5%, expression ≥ 88.5%, Dako Score ≥ 2, and other standards. In addition, due to the lack of information or inconsistent classification criteria in the literature, variables such as age and racial pathological type were not able to be combined and analyzed. In each case,heterogeneity was detected. Furthermore, a large part of the studies were retrospective, and more prospective studies are required to establish the causal relationship between EpCAM and prognostic indicators.

To overcome drug resistance in radiotherapy and chemotherapy as well as recurrence and metastasis, destroying CSCs, while shrinking the tumor has become an important strategy (102). They are a key area of tumor research and an important target for future cancer treatment (7). Researches on CSCs and their biomarkers is highly significant for the advancement of precision medicine. Precision medicine refers to treatments that differentiating a particular patient from other individuals exhibiting similar clinical manifestations according to genetic, biomarker, phenotype, or psychosocial characteristics, aimed at the needs of individual patients (103). The development of relevant therapies based on biomarkers targeting CSCs is a promising strategy for precise medical treatment and for reducing radiochemotherapy resistance, recurrence, and metastasis in patients with tumors.

Our results clearly demonstrated that the overexpression of EpCAM is an unfavorable prognostic indicator of OS and DFS in solid tumors, especially in gastrointestinal tumors. And EpCAM overexpression was related to the clinicopathological characteristics of solid tumors, particularly worse differentiation. EpCAM,with complex biological characteristics, serves as a promising candidate molecule for solid tumor detection and therapy. Further experimental and clinical researches are expected to reveal the mechanism by which EpCAM is conducive to the occurrence and development of solid tumors and to apply the biological characteristics of EpCAM to diagnosis and treatment.

## Data availability statement

The original contributions presented in the study are included in the article/supplementary material. Further inquiries can be directed to the corresponding authors.

## Author contributions

Research conception and design: PD, PC, JO, QL, and SL. Acquisition of data: PD, PC, and JO. Analysis of data: PD and PC. Drafting of the manuscript: PD and PC. Critical revision of the manuscript for important intellectual content: QL and SL. Statistical analysis: PD and PC. Supervision: QL and SL. All authors contributed to the article and approved the submitted version.
